# Beyond neuroprotection: Carbon monoxide–induced oligodendrogenesis and cognitive recovery

**DOI:** 10.4103/NRR.NRR-D-25-00550

**Published:** 2025-09-29

**Authors:** Shintaro Kimura, Josephine Lok, Ken Arai

**Affiliations:** Neuroprotection Research Laboratories, Departments of Radiology and Neurology, Massachusetts General Hospital and Harvard Medical School, Charlestown, MA, USA

**Carbon monoxide — from toxicity to therapeutic potential:** Carbon monoxide (CO) has long been known as a toxic gas, primarily associated with environmental pollution and poisoning. Its strong affinity for hemoglobin causes the formation of carboxyhemoglobin, which reduces oxygen delivery to the tissues and organs and leads to hypoxia. Despite its well-documented toxicity, previous studies have confirmed that CO also acts as a signaling molecule in the body and plays important physiological roles (Motterlini and Otterbein, 2010). This dual nature, both harmful and potentially beneficial, has led to studies of controlled CO exposure in the treatment of central nervous system (CNS) injuries.

CO is produced naturally in the body when heme is broken down by heme oxygenases (HO-1 and HO-2) to form CO, biliverdin, and ferrous iron, which together help regulate cellular homeostasis. HO-1, an enzyme activated in response to oxidative stress and inflammation, has been implicated in neuroprotection and disease processes. HO-2, which is constantly present in the CNS, contributes to neurovascular regulation, indicating that CO is involved in maintaining normal brain function. At low concentrations, CO may have several beneficial effects on cellular and molecular pathways. First, CO has anti-inflammatory properties, suppressing pro-inflammatory cytokines and modulating immune responses, making it a promising option for reducing neuroinflammation in CNS disorders. Second, CO promotes vasodilation by activating soluble guanylate cyclase, leading to increased production of cyclic guanosine monophosphate, which dilates blood vessels and improves oxygen delivery to neurons. Third, CO has anti-apoptotic effects; it helps prevent excessive programmed cell death by stabilizing mitochondrial survival pathways, allowing neurons to survive for a longer period. Fourth, CO may preserve circadian rhythms by modulating the expression of circadian genes, such as Per2. Finally, CO acts as a neuroprotective agent by reducing oxidative stress and increasing neuronal resilience after injury. Unlike traditional neuroprotective agents that act primarily within neurons, CO also affects blood vessels, glial cells, and immune responses, all of which are critical for CNS repair.

To safely utilize CO as a therapeutic substance while minimizing its toxic effects, scientists have developed carbon monoxide releasing molecules (CORMs), synthetic compounds designed to release CO in a controlled manner (Kautz et al., 2016; **Table 1**). These molecules provide a gradual and localized release of CO, reducing the risks associated with direct CO inhalation and making CO-based therapies more practical. Several types of CORMs have been developed for specific medical applications. Metal carbonyl-based CORMs, which contain metals such as ruthenium or manganese, stabilize CO and release it under certain conditions, providing predictable pharmacokinetics. Organic CORMs, which do not contain metals, offer better biocompatibility but require further optimization of their release mechanisms. A newer category, light-activated CORMs, release CO in response to ultraviolet or visible light, enabling precise spatial and temporal CO delivery, which may be beneficial for targeted CNS therapies. In addition, other CO-releasing scaffolds, including nanoparticle-based systems, may offer novel opportunities for highly targeted neuroprotective therapies, potentially paving the way for the next generation of CO-based treatments for CNS disorders. However, the selection of the optimal CORM formulation may depend on several factors, including the target tissue, CO release kinetics, and overall stability.

**Table 1:** Types and characteristics of CORMs

Types	Structural features	Advantages	Limitations/challenges
Metal carbonyl-based CORMs	Contain transition metals (e.g., Ru, Mn)	High chemical stability; controllable CO release under specific conditions	Potential biocompatibility and toxicity concerns
Organic CORMs	Metal-free organic compounds	Better biocompatibility	Optimization of CO release mechanisms still needed
Light-activated CORMs	Activated by ultraviolet or visible light	Enable spatiotemporal control of CO delivery	Application to deep tissue (e.g., CNS) requires special light delivery methods
Nanoparticle-based CORMs	CO release integrated into nanoparticle carriers	Allow targeted delivery and potentially enhanced specificity	Further development needed for clinical application

CNS: Central nervous system; CO: carbon monoxide; CORMs: carbon monoxide releasing molecules.

**Roles of CO in CNS disease treatment — a multi-faceted approach:** Because of its diverse biological effects, CO may have therapeutic potential for CNS disorders, including stroke, spinal cord injury, and several neurodegenerative diseases (Queiroga et al., 2015). CO provides neuroprotection by targeting secondary injury cascades, i.e., the molecular and cellular processes that exacerbate neurological damage after an initial injury. One of the most important of these mechanisms is neuroinflammation, a key feature of many CNS disorders. Following brain injury, microglia and astrocytes become activated and release pro-inflammatory cytokines such as tumor necrosis factor-α, interleukin-6, and interleukin-1β. This inflammatory response plays a major role in neuronal damage. CO has been shown to inhibit excessive microglial activation, thereby shifting the immune reaction to a more protective state by modulating the balance between pro- and anti-inflammatory responses. As a result, CO therapy may help reduce the deleterious effects of neuroinflammation. In addition, CO activates the HO-1 pathway, which stimulates the production of biliverdin and bilirubin, two molecules with potent anti-inflammatory and antioxidant properties. These effects not only suppress inflammation, but also reduce oxidative stress, a key factor in the progression of neurodegenerative diseases. By helping to balance pro- and anti-inflammatory signaling, CO therapy may offer a regulated immunomodulation strategy for CNS disorders.

Beyond its role in modulating inflammation, CO also plays an important role in supporting vascular health (Motterlini and Otterbein, 2010). Maintaining proper blood flow is critical for maintaining brain function, as neurons require a continuous supply of oxygen and glucose through the cerebrovascular network. CO promotes vasodilation by interacting with soluble guanylate cyclase, which triggers the production of cyclic guanosine monophosphate. Higher levels of cyclic guanosine monophosphate relax blood vessels and improve cerebral blood flow, an important limiting factor in conditions such as stroke and vascular dementia. In addition, CO protects endothelial cells by reducing vascular permeability and preventing damage to the blood–brain barrier (BBB). A weakened BBB is common in neurodegenerative diseases, allowing harmful molecules and inflammatory agents to enter the brain and exacerbate neuronal damage. The ability of CO to maintain BBB integrity suggests a potential role in long-term neuroprotection and slowing of progressive neurological decline. In addition to its effects on vascular integrity, CO also counteracts oxidative stress, another major contributor to CNS disorders, including stroke, Alzheimer’s disease, and amyotrophic lateral sclerosis (Queiroga et al., 2015; Li et al., 2022). Excessive accumulation of reactive oxygen species leads to protein aggregation, mitochondrial dysfunction, and neuronal apoptosis, all of which accelerate disease progression. CO helps counteract oxidative damage by activating antioxidant defense mechanisms, particularly the Nrf2 pathway, which increases the production of antioxidant enzymes, such as glutathione peroxidase and catalase. In addition, CO affects mitochondrial activity by preventing excessive cytochrome c release, a key step in the initiation of apoptosis. By stabilizing mitochondrial function, CO therapy may support neuronal survival under stressful conditions, potentially reducing neurodegeneration and slowing disease progression.

**Oligodendrogenesis and cognitive recovery — expanding the scope of CO therapy in traumatic brain injury:** Traumatic brain injury (TBI) is one of many CNS disorders for which CO therapy has demonstrated neuroprotective potential in preclinical studies (Choi et al., 2016). Because CNS disorders range from acute injuries such as stroke and spinal cord trauma to chronic diseases such as Alzheimer’s disease and vascular dementia, effective treatment strategies would need to address multiple pathological processes, at times simultaneously. The ability of CO to modulate inflammation, oxidative stress, and vascular function makes it a promising candidate for neuroprotection and functional recovery in CNS injury.

Accumulating evidence on CO therapy for CNS injury has deepened our understanding of its neuroprotective mechanisms, and our recent study introduced a new aspect of CO-based interventions – exogenously administered CO promoted brain repair and recovery after TBI by increasing oligodendrogenesis, a process that has received relatively little attention in the context of TBI recovery (Choi et al., 2025). One of the novel findings of the study is the role of A-kinase anchor protein 12 (AKAP12) in oligodendrocyte regeneration during CO therapy (CORM3 treatment). Previously recognized as a scaffolding protein involved in signal transduction, AKAP12 is now proposed to play an important role in myelin repair and functional recovery after brain injury (Kimura et al., 2023). Our data suggest that CO therapy upregulates AKAP12 expression, which enhances oligodendrocyte maturation and improves myelin integrity in the injured brain. Notably, AKAP12 knockout mice failed to show the therapeutic benefits of CO; they did not exhibit increased oligodendrogenesis and cognitive recovery when given CORM3. This suggests that AKAP12 is essential for effective neuronal repair in TBI after CO therapy. In addition, the study shows that CO therapy not only preserves existing oligodendrocytes, but also actively stimulates the generation of new oligodendrocytes, further adding to its critical role in facilitating recovery after TBI.

Another important finding of this study is the identification of pericytes as key regulators of CO-mediated oligodendrogenesis. Traditionally known for their role in maintaining vascular and BBB integrity, pericytes have now been found to directly contribute to neuroregenerative processes (Sweeney et al., 2016). Specifically, results show that in response to CORM3 treatment, AKAP12-expressing pericytes help to create a supportive microenvironment for oligodendrocyte survival and myelination. This pericyte-oligodendrocyte pathway may provide new insights into how vascular and glial interactions drive neurorepair and extend the conventional understanding of pericyte function beyond its role in vascular regulation. By stimulating pericyte-derived AKAP12 signaling, CO therapy provides support for the differentiation of oligodendrocyte precursor cells into mature oligodendrocytes, which then integrate into neuronal circuits, highlighting the importance of vascular-glial crosstalk in CNS repair.

In addition to the molecular and histological findings, this study also provides behavioral evidence supporting the efficacy of CO therapy. Wild-type mice treated with CO therapy showed significant improvements in cognitive function as assessed by post-TBI behavioral tests. In contrast, AKAP12-deficient mice failed to show cognitive recovery, highlighting the role of AKAP12-driven oligodendrogenesis in functional recovery after TBI. These results establish a direct link between glial regeneration and behavioral recovery and underscore the fact that successful oligodendrogenesis may contribute to long-term neurological improvement after TBI.

**Conclusions & future remarks — advancing CO therapy for CNS repair:** Research on CO therapy is expanding our understanding of CNS recovery. Historically, TBI recovery strategies have focused on neurogenesis and neuronal survival, but an integrative approach that includes oligodendrocyte development and myelin reconstruction is needed for a successful outcome. The study introduced above focused on AKAP12 and oligodendrogenesis in CO therapy for TBI and demonstrated that effective recovery requires not only neuronal protection, but also glial regeneration and myelin repair. The identification of AKAP12 as an important mediator of CO-induced oligodendrogenesis introduces a key signaling pathway that may serve as the basis for future neuroregenerative therapies. In addition, the role of pericytes in oligodendrocyte maturation provides a novel therapeutic avenue and highlights the importance of neurovascular interactions in repair after injury.

**Figure 1:**
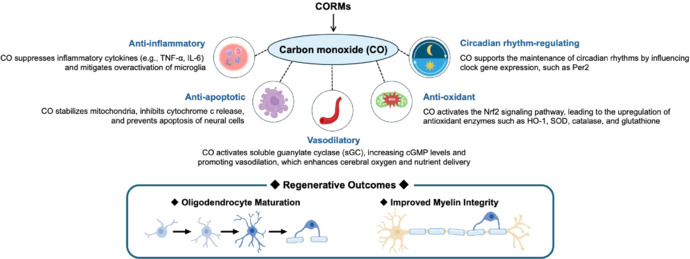
Beneficial effects of CO in the CNS. This schematic illustrates the multifaceted roles of CO in cellular and molecular pathways relevant to CNS disorders. At low concentrations, CO may function as both a neuroprotective and neuroregenerative agent by reducing oxidative stress and enhancing tissue resilience. cGMP: Cyclic guanosine monophosphate; CNS: central nervous system; CO: carbon monoxide; CORMs: carbon monoxide releasing molecules; HO-1: heme oxygenase 1; IL-6: interleukin 6; SOD: superoxide dismutase; TNF: tumor necrosis factor.

This perspective highlights the importance of neuroprotective mechanisms of CO, particularly its ability to regulate vascular function, modulate inflammation, and counteract oxidative stress, in addition to promoting oligodendrocyte maturation and myelin integrity (**Figure 1**). Despite its promise, clinical translation of CO therapy remains challenging. Optimization of delivery mechanisms is critical, as current CORMs exhibit variations in release kinetics and tissue specificity that require refinement to ensure safety and efficacy in CNS applications. In addition, the long-term effects of CO exposure on neural (or brain) health require rigorous evaluation, as prolonged treatment of CORMs may pose potential neurotoxicity risks. The establishment of standardized dosing strategies is also essential to ensure both efficacy and safety in various CNS disorders. Looking ahead, several key research directions could accelerate the integration of CO therapy into clinical applications. Expanding studies beyond TBI to include stroke, spinal cord injury, and neurodegenerative diseases would provide a broader understanding of the therapeutic potential of CO. Further investigation of AKAP12 signaling in pericytes and other types of brain cells may also reveal novel neuroregenerative mechanisms. In addition, CO modulates circadian rhythms, which affect the severity of brain damage in spontaneous intracerebral hemorrhage in mice (Schallner et al., 2017) and impact oligodendrocyte functions that are necessary for brain recovery after injury (Rojo et al., 2023). Future investigations into the molecular effects of CORMs on genes and proteins related to circadian rhythms and oligodendrocyte function are warranted, to elucidate the interactions among CO signaling, circadian rhythms, and compensatory oligodendrogenesis. Finally, the combination of CO therapy with established neuroprotective agents (Takase et al., 2018) or the integration of CO therapy with non-pharmacological interventions, such as exercise (Kinoshita et al., 2022), may have synergistic effects and potentially enhance neuronal and glial recovery. By overcoming current translational challenges and refining delivery methods, CO therapy holds great promise as a viable clinical intervention, offering new opportunities for patients suffering from CNS injury.

*The authors thank Dr. Eng H. Lo, Dr. Yoon Kyong Choi, and other members of the Neuroprotection Research Laboratories at Massachusetts General Hospital for their advice on this manuscript.*

*This work was supported in part by the NIH (R01NS113556, to KA).*
